# Novel Sensors Based on the Symmetry Properties of Split Ring Resonators (SRRs)

**DOI:** 10.3390/s110807545

**Published:** 2011-07-29

**Authors:** Jordi Naqui, Miguel Durán-Sindreu, Ferran Martín

**Affiliations:** CIMITEC, Departament d’Enginyeria Electrònica, Universitat Autònoma de Barcelona, Bellaterra, Barcelona 08193, Spain; E-Mails: miguel.duransindreu@uab.cat (M.D.-S.); ferran.martin@uab.cat (F.M.)

**Keywords:** split ring resonators, metamaterials, coplanar waveguide, position sensors, angle sensors

## Abstract

The symmetry properties of split ring resonators (SRRs) are exploited for the implementation of novel sensing devices. The proposed structure consists of a coplanar waveguide (CPW) loaded with movable SRRs on the back substrate side. It is shown that if the SRRs are placed with the slits aligned with the symmetry plane of the CPW, the structure is transparent to signal propagation. However, if the symmetry is broken, a net axial magnetic field can be induced in the inner region of the SRRs, and signal propagation is inhibited at resonance. The proposed structures can be useful as alignment sensors, position sensors and angle sensors. This novel sensing principle is validated through experiment.

## 1. Introduction

**Figure 1.**
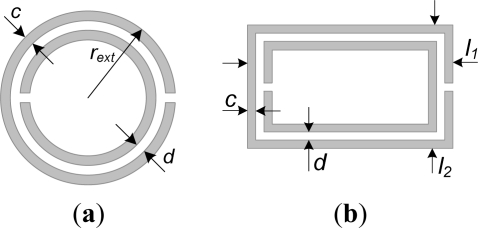
Typical topology and relevant dimensions of (**a**) a circular and (**b**) a rectangular SRR.

Split ring resonators (SRRs) consist of a pair of concentric metallic rings, etched on a dielectric substrate, with slits etched on opposite sides (see Figure 1). SRRs can be electrically small if the rings are tightly coupled [1]. These resonators have been used for the synthesis of left handed [2] and negative refractive index media [3], where the necessary value of the negative effective permeability is due to the presence of the SRRs (if an array of electrically small SRRs is excited by means of an axial time varying magnetic field, the structure behaves as an effective medium with negative effective permeability in a narrow band above SRR resonance). SRRs have also been coupled to planar transmission lines, for the synthesis of transmission line metamaterials. Specifically, it was demonstrated in [4] that a coplanar waveguide (CPW) loaded with pairs of SRRs in the back substrate side inhibits signal propagation in the vicinity of SRR resonance, and this was interpreted as due to the negative effective permeability of the structure. Indeed, if a CPW is loaded with a single pair of SRRs, the transmission coefficient exhibits a transmission zero, indicative of the resonance frequency of the SRRs. Such resonance frequency is given by the inductance and capacitance of the SRR, where a quasi-static model can be applied if the particle is electrically small [5]. Thus, for electrically small circular SRRs, the inductance is calculated from the inductance of a single ring with average radius, and the capacitance is given by the series connection of the distributed capacitance of the upper and lower halves of the SRR [5]. Actually, the SRR exhibits many resonance frequencies [6], but the frequency of interest for the implementation of effective media is the first one, because the particle can only be considered to be electrically small at this resonance.

Different kind of sensors based on SRRs have been proposed, such as thin-film sensors [7], particle detectors [8,9], stress sensors [10], moisture sensors [11], pressure sensors [12], or displacement sensors [13], among others. In most of these applications, the variation of the resonance frequency or the quality factor of the particles, caused by changes in the variable to be sensed, has been the considered sensing strategy. In this paper, a different approach for the implementation of sensors based on SRRs is proposed, *i.e.*, to exploit the symmetry properties of SRRs coupled to CPWs. The detection principle is based on the loss of symmetry caused, for instance, by a displacement or a rotation.

In Section 2, the theory behind the proposed detection principle is explained in detail; in Section 3, the principle is validated through electromagnetic simulation; the sensitivity of a prototype device is evaluated through simulation and experiment in Section 4 to demonstrate the proof-of-concept; finally, the main conclusions of the work are highlighted in Section 5.

## 2. The Sensing Principle

**Figure 2.**
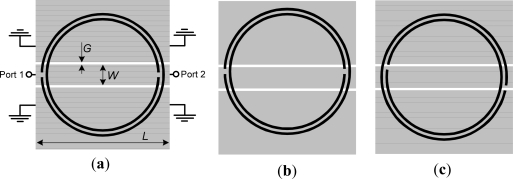
CPW loaded with a circular shaped SRR; (**a**) symmetrically placed; (**b**) displaced 0.5 mm; and (**c**) rotated 10°. Dimensions are: *L* = 10.4 mm, *W* = 1.67 mm, *G* = 0.2 mm, *c* = *d* = 0.2 mm and *r_ext_* = 5 mm.

As reported in [4], a CPW structure loaded with pairs of SRRs inhibits signal propagation in the vicinity of SRR resonance. In the reported prototypes, the pairs of SRRs are etched on the back substrate side, with the center of the SRRs underneath the slots. Under these conditions, the magnetic field in the inner region of each SRRs is contradirectional, but the SRRs can be excited at resonance. Let us consider, instead, that only one SRR is etched in the back substrate side of a CPW structure, and that the slit of each ring is perfectly aligned with the symmetry plane of the CPW structure [see Figure 2(a)]. Under these conditions, it is clear that the axial components of the magnetic field in the inner region of the SRR must cancel, and the SRR cannot be excited at resonance. However, if the symmetry is broken, a net magnetic field in the inner region of the SRR is expected and the rings will be excited at resonance. This situation can be simply monitored by measuring the transmission coefficient. A transmission zero at SRR resonance is expected, with the magnitude of such transmission zero being dependent on the deviation from symmetry (the considered sensitivity in this work is thus given by the derivative of the notch magnitude with the variable to be sensed, that is, lateral displacement or rotation).

Alternatively, the fact that the SRR is not excited if the slits are perfectly aligned with the symmetry plane of the CPW can be explained since the symmetry plane of the SRR is an electric wall at resonance [5], *i.e.*, the distribution of charges is anti-symmetric with respect to that plane. However, the symmetry plane of a CPW structure is a magnetic wall for the fundamental (even) mode. Thus, the SRR cannot be excited if both symmetry planes (that of the CPW and that of the SRR) coincide.

## 3. Validation

**Figure 3.**
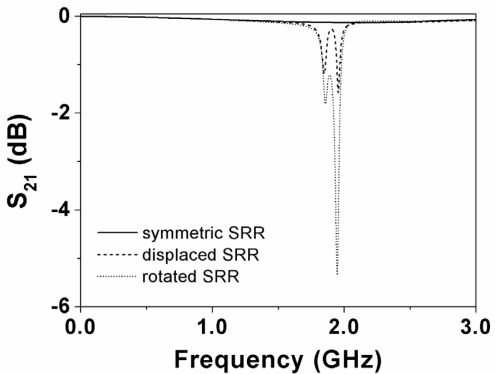
Simulated transmission coefficients of the structures of Figure 2.

In order to validate the previous principle, we have considered a CPW loaded with a circular SRR (Figure 2) in three configurations: (a) symmetrically etched; (b) displaced 0.5 mm; and (c) rotated 10°. Although the lack of symmetry can be produced by many different causes, in this work we have considered the following two possibilities: a lateral displacement and a rotation of the SRR. The resonance frequency of the SRR has been estimated by means of the model reported in [5] (*f*_0_ = 1.94 GHz), by considering the Rogers RO3010 substrate with dielectric constant *ε_r_* = 10.2, thickness *h* = 127 μm, and loss tangent tan*δ* = 0.0023. In these structures, we have obtained the transmission coefficient (*S_21_*) by means of a full wave electromagnetic simulator (Agilent Momentum). The results, shown in Figure 3, reveal that if the SRR is symmetrically etched, the structure is transparent at SRR resonance. However, if the symmetry is altered by simply displacing or rotating the SRR, a notch appears in the transmission coefficient at the resonance frequency of the SRR. It is worth to mention that actually, in the current validation, there are two notches which are the corresponding ones to the inner and outer ring. It has been observed that they are very close because the rings have nearly the same length and are loosely coupled due to the thin substrate thickness (a short distance between the SRR and the top layer, with respect to the gap between rings, favors the coupling between the top layer and the SRR at the expense of the inter-coupling between rings). Notice that the substrate thickness (127 μm) is thinner than the inter-ring separation (200 μm), a value close to the fabrication limit. If the two rings forming the SRRs are loosely coupled, the SRR cannot be considered to be electrically small. Hence, the first resonance frequency is no longer quasi-static, but the symmetry properties and sensing principle described in the previous section are exactly the same. Thus, it is clear that we are able to detect the lack of symmetry in CPWs loaded with a single SRR. The effects of substrate thickness on inter-rings coupling are analyzed later, on the basis of the structures considered to validate the sensing principle.

It is important to obtain a high sensitivity of the notch magnitude with the variable that destroys the symmetry of the structure. Obviously the sensitivity is expected to be dependent on the SRR and CPW geometry, but we do also expect the influence of substrate thickness. In structures with thick substrates, the magnetic field lines generated by current flow in the CPW scarcely penetrate the SRR region and hence we do not expect a high sensitivity under these conditions. Therefore, CPW structures with thin substrates are preferred for the design of sensors. Among the commercially available low-loss microwave substrates of our laboratory, we have thus chosen the Rogers RO3010 with thickness *h* = 127 μm.

**Figure 4.**
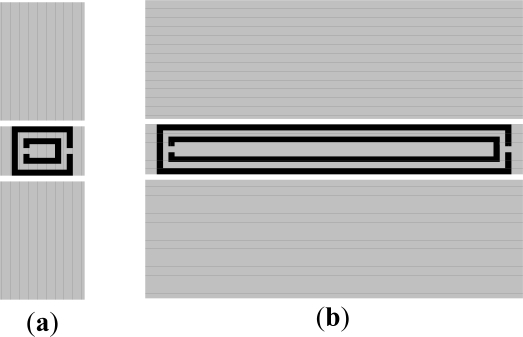
(**a**) CPW loaded with a square shaped SRR; and (**b**) CPW loaded with a rectangular shaped SRR. Dimensions are: *L_1_* = 2.07 mm (**a**), *L_1_* = 12.4 mm (**b**), *W* = 1.67 mm, *G* = 0.2 mm, *c* = *d* = 0.2 mm, *l_1_* = 1.67 mm (**a**), *l_1_* = 12 mm (**b**) and *l_2_* = 1.67 mm.

**Figure 5.**
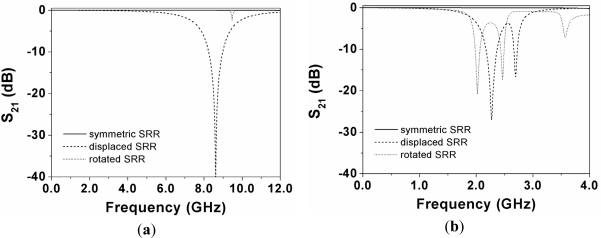
Simulated transmission coefficients of the structures of Figure 4 and those obtained by displacing 0.5 mm and rotating 10° the SRR; (**a**) Figure 4(a) and (**b**) Figure 4(b).

With regards to the geometry, by decreasing the overall dimensions of the CPW and SRR, the sensitivity for a linear displacement can be enhanced. The reason is that if we scale down the structure and a particular displacement, the frequency response experiences a shift upwards roughly maintaining the same notch magnitude (such notch magnitudes being identical if losses are neglected). Nevertheless, the limits are dictated by the available fabrication technology (a photo mask/etching process with a minimum strip and slot width of 200 μm in our case). The critical aspect to enhance sensitivity is to choose the appropriate shape factor for the SRR. Namely, if we choose a circular SRR with an internal diameter much larger than the distance between the CPW slots (as in Figure 2), the magnetic field lines within the SRR region do not exactly cancel if symmetry is broken, but the effects of asymmetry are not significant. Conversely, if the SRR does not extend beyond the slots of the CPW, the effects of the asymmetry on the frequency response of the structure (notch magnitude) are more pronounced. Indeed, the magnitude of the notch increases by etching the SRR just below the central strip of the CPW. To validate the previous comments we have considered three different SRR-loaded CPW structures. One of them is that depicted in Figure 2(a); the others are those depicted in Figure 4. In all these structures, the symmetry has been broken by applying a lateral displacement and by applying a rotation to the SRR of 0.5 mm and 10°, respectively. The simulated transmission coefficients obtained after applying the previous translation and rotation operations to the SRR are depicted in Figure 3 and in Figure 5. According to these figures, the structure of Figure 4(a) provides the deepest notch for the displacement, whereas the structure of Figure 4(b) seems to be the best choice for detecting rotation. It is also remarkable that although for the square-shaped SRR the rings are uncoupled (as for the circular and rectangular shaped SRRs), the notches are substantially distant because the lengths of the inner and outer ring are very different.

**Figure 6.**
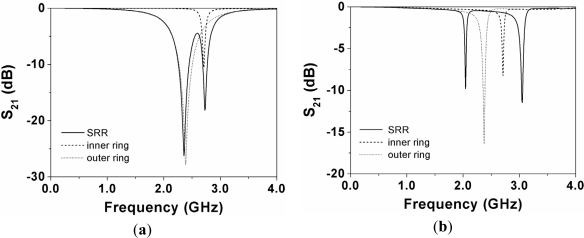
Simulated transmission coefficients of the structure of Figure 4(b) by laterally displacing 0.5 mm the SRR, and by considering only the inner or the outer ring (also displaced); (**a**) *h* = 127 μm and (**b**) *h* = 635 μm.

To gain more insight on the effects of the substrate thickness on inter-resonator’s coupling, the frequency response of the CPW with the individual rings of Figure 4(b) laterally displaced 0.5 mm has been simulated. The results are compared with the frequency response of the SRR-loaded CPW in Figure 6(a). Clearly, the rings are uncoupled. However, if substrate thickness is increased to 635 μm [Figure 6(b)], the first two resonances of the SRR experience a frequency shift caused by an evident inter-rings coupling. However, the depth of the notches decreases; thus thin substrates are preferred, even though this can result in a negligible inter-rings coupling.

## 4. Experimental Results

The purpose of this section is to analyze the sensitivity of the proposed sensing devices by means of full wave electromagnetic simulation and experiment. To this end, rather than fabricating a sensing device with movable SRRs, which is technologically more complex, we will study the effects of displacing or rotating the SRRs by fabricating several devices with different displacements and rotations (the key point of this paper is to validate the proof-of-concept for this novel sensing principle).

**Figure 7.**
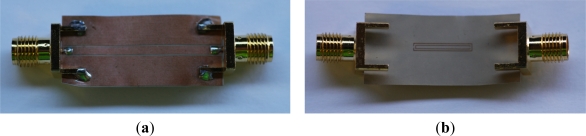
Photograph of the fabricated base structure; (**a**) top view of the CPW; and (**b**) SRR etched in the back substrate side.

**Figure 8.**
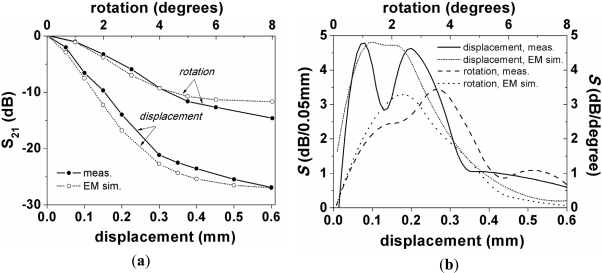

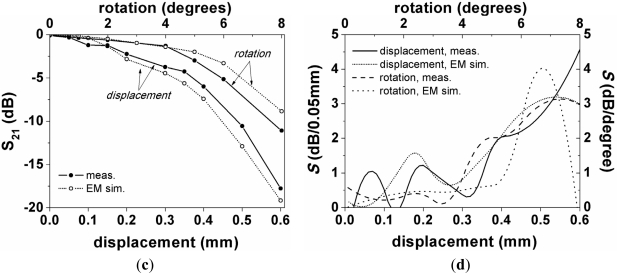
(**a**) Insertion loss at first resonance and (**b**) sensitivity of the insertion loss magnitude for the prototype device shown in Figure 7, resulting by laterally displacing and by rotating the SRR. In (**c**) and (**d**), the results for the second resonance are depicted. The measured frequency responses, necessary to obtain the magnitude of the first and second notch, have been obtained by means of the Agilent E8364B vector network analyzer.

The base structure (*i.e.*, the symmetric structure) is depicted in Figure 7. In such structure, the slot and central strip widths of the CPW are *G* = 0.2 mm and *W* = 1.67 mm, respectively (corresponding to a 50 Ω line in the Rogers RO3010 substrate indicated before). The width, *c*, of the metal strips of the SRR as well as their separation, *d*, are *c* = *d* = 0.2 mm; finally, the length and width of the SRR is *l_1_* = 12 mm and *l_2_* = 1.67 mm, respectively. These dimensions have been optimized to enhance the sensitivity, taking into account the minimum strip and slot width of the available technology and the effects of losses. From this structure, we have fabricated some additional CPWs with different displaced and rotated SRRs. For these structures, we have obtained the magnitude of the insertion loss at the first and second resonance; the results are depicted in Figure 8. There is a reasonable agreement between the electromagnetic simulations and the measurements. The sensitivity *S* (Δ*S_21_*/Δ*x* and Δ*S_21_*/Δ*φ*, where *x* and *φ* denote displacement and rotation, respectively), simply inferred from the discrete derivative of the insertion loss magnitude (after applying a smoothing algorithm), is also depicted in the figure. The results are reasonable for the first resonance, taking into account the dimensions of the considered SRR and CPW. For the second resonance, the sensitivity is not as good as the one corresponding to the first resonance, the reason being that the ring producing the second resonance is smaller and further displacement is required to notice the effects of asymmetry (*i.e.*, the inner ring needs more lateral displacement to reach the slot area of the CPW).

**Figure 9.**
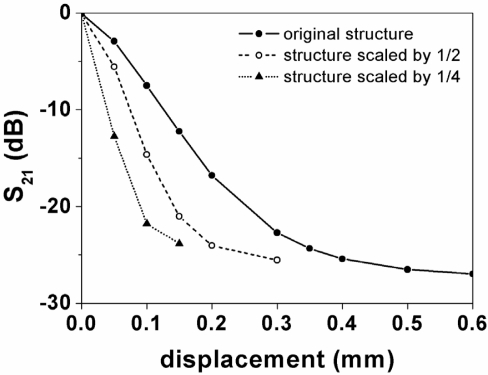
Comparison between the notch depth given by electromagnetic simulation for the structure of Figure 7 and for the same structure with all dimensions scaled down.

To end this section, the notch magnitude (first resonance) obtained by electromagnetic simulation of the structure of Figure 7 that results from lateral displacement of the SRR is compared (Figure 9) to that of an identical structure but with all dimensions scaled down by a factor of two and four (dielectric and ohmic losses are included in the simulations). As can be seen, sensitivity is enhanced by scaling dimensions down, the improvement being nearly proportional to the scaling factor. However, as the scaling factor increases losses play an increasingly significant role and, for a too large scaling factor, sensitivity might be lower than expected. From the results of this section, it can be inferred that the sensing principle is validated.

## 4. Conclusions

In conclusion, a new sensing principle for the detection of displacement and rotation, based on the loss of symmetry in SRR-loaded CPW structures, has been validated through theory and experiment. It has been shown that thin substrates and square/rectangular shaped SRR are necessary to enhance the sensitivity of the structures. A set of structures with different laterally shifted and rotated SRRs have been fabricated to validate the sensing principle. The resulting sensitivity of these structures exhibits a maximum measured value of roughly 95 dB/mm and 3.4 dB/degree for the displacement and angle sensor (first resonance), respectively.
